# Apolipoprotein ε4 exacerbates white matter impairment in a mouse model of Aβ amyloidosis by decreasing actively myelinating oligodendrocytes

**DOI:** 10.1002/alz.70791

**Published:** 2025-10-11

**Authors:** Md Mamun Al‐Amin, Byungwook Kim, Hande Karahan, Mason D. Tate, Sam P. Walsh, Shweta S. Puntambekar, Stephanie J. Bissel, Bruce T. Lamb, Nian Wang, Jungsu Kim

**Affiliations:** ^1^ Stark Neurosciences Research Institute Indiana University School of Medicine Indianapolis Indiana USA; ^2^ Department of Medical and Molecular Genetics Indiana University School of Medicine Indianapolis Indiana USA; ^3^ Medical Neuroscience Graduate Program Indiana University School of Medicine Indianapolis Indiana USA; ^4^ Radiology and Imaging Sciences Indiana University School of Medicine Indianapolis Indiana USA

**Keywords:** Alzheimer's disease, apolipoprotein E4, myelin, oligodendrocyte, white matter

## Abstract

**INTRODUCTION:**

The ε4 allele of the apolipoprotein E (*APOE*) gene is a risk factor for the development of Alzheimer's disease (AD). *APOE4* isoform is associated with increased white matter lesions in humans. To identify the underlying mechanisms of white matter impairment associated with *APOE4*, we investigated the effects of *APOE4* and *APOE3* on multiple readouts of the white matter microstructural integrity.

**METHODS:**

Using magnetic resonance imaging and immunohistochemistry approaches, we analyzed white matter tracts in 5xFAD mice expressing *APOE3* (5xFAD;*APOE3*) or *APOE4* (5xFAD;*APOE4*).

**RESULTS:**

*APOE4* significantly decreased fractional anisotropy, axial diffusivity, and neurite density index, while increasing radial diffusivity and isotropic volume fraction within major white matter tracts. Myelination was reduced in the corpus callosum of 5xFAD;*APOE4* mice. Mechanistically, *APOE4* reduced populations of mature and actively myelinating oligodendrocytes.

**DISCUSSION:**

Our results suggest that a decrease in the number of actively myelinating oligodendrocytes may explain myelin loss, leading to white matter impairments.

**Highlights:**

- A robust neurite orientation dispersion and density imaging (NODDI) approach to study the effect of apolipoprotein E (APOE) isoforms on the white matter in 5xFAD mice.
- APOE4 reduces neurite density and increases water accumulation in the white matter.
- APOE4 disrupts structural connectivity and reduces the betweenness centrality.
- APOE4 decreases the number of actively myelinating oligodendrocytes.
- A reduction in myelinating oligodendrocyte populations may lead to myelin loss.

## 1 BACKGROUND

Alzheimer's disease (AD) is characterized by progressive cognitive decline and the accumulation of amyloid‐β (Aβ) and tau in the brain. The apolipoprotein E (*APOE*) gene plays a critical role in AD pathogenesis, with its variants (*APOE2*, *APOE3*, and *APOE4*) differentially influencing Aβ metabolism and other pathways.^1^, ^2^, ^3^ Among these, *APOE2* is generally considered protective against AD,^4^ whereas *APOE3* is regarded as neutral in terms of disease risk. Conversely, *APOE4* is a well‐established risk factor for AD, increasing disease susceptibility compared with *APOE3*.^5^ The impact of *APOE4* on cortical and subcortical structures has been well documented.^6^, ^7^ Patients with two copies of the *APOE4* allele exhibit atrophy in the hippocampus and the entorhinal cortex, regions critical for memory and cognitive function.^6^, ^8^, ^9^ Beyond its effects on gray matter, *APOE4* also compromises the integrity of white matter, further disrupting brain connectivity and function.^10^, ^11^, ^12^ Therefore, understanding the mechanisms behind these phenotypes is crucial for developing therapeutic strategies.

In AD, damage to white matter has been associated with impaired executive function and weakened memory performance.^13^ Impaired white matter integrity is a hallmark of the early stage of AD,^14^, ^15^ and it is thought that tau and Aβ deposition contribute to the loss of white matter integrity.^16^, ^17^, ^18^ Microstructural damage in the white matter of AD patients has been reported in the corpus callosum, fornix, and cingulum.^19^, ^20^, ^21^ This damage is associated with disrupted structural connectivity and impaired network functions.^19^, ^20^, ^21^

Multiple MRI studies have reported that *APOE4* isoform is associated with white matter degeneration in patients with dementia.^10^, ^11^, ^22^, ^23^ Even cognitively normal elderly *APOE4* carriers also exhibit an accelerated decline in white matter integrity.^12^ Myelin maintenance in white matter is actively regulated by the continuous proliferation and maturation of oligodendrocytes derived from progenitor cells throughout adulthood.^24^, ^25^ *APOE4* is known to disrupt oligodendrocyte differentiation and contribute to myelin breakdown in mouse models without any AD pathology.^26^, ^27^, ^28^ However, it remains unknown whether *APOE4* allele regulates mature oligodendrocytes in a mouse model of Aβ amyloidosis.

Actively myelinating oligodendrocytes are responsible for the ongoing formation and repair of myelin during the demyelinating process observed in multiple sclerosis and multiple system atrophy.^29^, ^30^ Given that demyelination is also observed in AD, investigating these actively myelinating oligodendrocytes in AD is critical to understand white matter impairment. However, it is unknown whether *APOE4* allele affects the actively myelinating oligodendrocytes in a mouse model of Aβ amyloidosis. This knowledge gap critically limits our understanding of the cellular mechanisms underlying *APOE4*‐driven white matter impairment in AD. To address this critical gap, we assess the effects of *APOE3* and *APOE4* on both mature and actively myelinating oligodendrocytes and white matter integrity in a mouse model of Aβ amyloidosis. We utilized both magnetic resonance imaging (MRI) and histological analyses with the same cohort, enabling direct correlations between in vivo imaging and underlying cellular alterations. Notably, previous studies have not combined MRI and histological investigation within the same subjects.^12^, ^22^, ^26^, ^27^ As a result, none have validated in vivo white matter abnormalities detected by MRI with corresponding cellular changes in tissues. To address this critical gap, we designed a unified experimental framework that analyzes both in vivo MRI scans and cellular alterations within the same samples.

### 1.1 Aim of the study

We investigated the effects of *APOE3* and *APOE4* isoforms on multiple measures of white matter integrity in 5xFAD mice. To the best of our knowledge, no prior in vivo studies have investigated how these *APOE* alleles influence white matter integrity and structural connectivity in a mouse model of Aβ amyloidosis using advanced neuroimaging techniques known as neurite orientation dispersion and density imaging (NODDI). Conventional diffusion‐weighted imaging (DWI) cannot accurately quantify microstructural properties of brain tissues with non‐Gaussian water diffusion, such as neurite density. In contrast, NODDI provides enhanced sensitivity and specific metrics for detecting AD‐associated microstructural changes.^31^, ^32^, ^33^

## 2 METHODS

### 2.1 Animals

A total of 23 mice (12–13 months old) were studied, including 12 5xFAD;*APOE3* (male = 9; female = 3) and 11 5xFAD;*APOE4* (male = 7; female = 4) mice. Mice with humanized *APOE3* (IMSR_JAX: 029018) and *APOE4* (IMSR_JAX: 027894) were crossed with 5xFAD transgenic mice^34^ (MMRRC stock #: 34848) to generate 5xFAD;*APOE3* and 5xFAD;*APOE4* mice. Humanized *APOE3* (*APOE3* ^+/+^) and *APOE4* (*APOE4* ^+/+^) knock‐in mice were generated by replacing the endogenous mouse *APOE* gene with human *APOE*3 or *APOE4*. Both strains were maintained on a C57BL/6J background. Next, we bred these *APOE* knock‐in mice with heterozygous 5xFAD^+/−^ transgenic mice to create experimental cohorts expressing a human *APOE* isoform (*APOE*3 or *APOE4*) alongside the 5xFAD mutations. The resulting offspring, 5xFAD^+/−^;*APOE3* ^+/+^ and 5xFAD^+/−^;*APOE4* ^+/+^, served as models to study the effects of APOE isoforms in the context of amyloid deposition. All the mice were housed at the Stark Neurosciences Research Institute under standard conditions and had ad libitum access to food and water. The mice were euthanized immediately after MRI data acquisition at 12–13 months of age. All animal experiments were approved and performed in compliance with the guidelines of the Institutional Animal Care and Use Committee at Indiana University (IACUC protocol number 24125).

### 2.2 Acquisition of structural and diffusion MRI data

Structural and diffusion MRI data were acquired using a 30‐cm bore 9.4T MRI scanner (Bruker BioSpec 94/30 Germany) at the Roberts Neuroscience Preclinical Imaging Facility, Stark Neurosciences Research Institute, Indiana University School of Medicine. A 2D T_2_‐weighted turbo sequence was employed to acquire the structural image using a rapid acquisition with relaxation enhancement (RARE) factor 8. The sequence parameters were as follows: slice thickness 300 µm, in‐plane resolution 75 × 75 µm, matrix size 200 × 172, field of view 20 × 17.2 mm, 54 continuous slices, repetition time 6200 ms, and echo time 32 ms.

Diffusion‐weighted images were acquired via a multishot 3D echo‐planar imaging (EPI) pulse sequence. The sequence parameters were as follows: matrix size 100 × 86, field of view 20 × 17.2 mm, 54 continuous slices, echo time 26.3 ms, repetition time 3500 ms, in‐plane resolution 150 × 150 µm, slice thickness 0.3 mm. A multishell diffusion scheme was employed with *b* values of 600, 1200, 1800, and 2400 s/mm^2^. The number of diffusion gradient directions acquired at each *b*‐value was 18, 18, 36, and 36. Two *b* = 0 images were acquired for each shell. A high‐sensitivity cryogenically cooled radiofrequency surface receive‐only coil (Bruker CryoProbe) was used to increase the signal‐to‐noise ratio.

**RESEARCH IN CONTEXT**

1. **Systematic review**: The authors reviewed the literature on the impact of the apolipoprotein ε4 (*APOE4*) allele on microstructural integrity in AD through a PubMed search. No robust animal study has used neurite orientation dispersion and density imaging, a technique more sensitive than diffusion imaging for detecting white matter pathology.
2. **Interpretation**: This study reveals that *APOE4* allele exacerbates white matter deficits in an amyloid mouse model. Key findings include decreased fractional anisotropy, axial diffusivity, and neurite density index, alongside increased radial diffusivity and isotropic volume fraction. *APOE4* mice exhibit a reduced number of actively myelinating oligodendrocytes, likely leading to myelin loss and white matter damage. These findings suggest a link between oligodendrocyte dysfunction and *APOE4‐*driven pathology.
3. **Future directions**: These findings highlight the need for future studies to explore strategies for replenishing actively myelinating oligodendrocytes, offering novel therapeutic approaches to mitigate *APOE4*‐associated white matter degeneration in AD.

We performed in vivo MRI scans when the animals were 12–13 months old. This time point represents advanced stage of Alzheimer's‐like pathology and functional decline in the 5xFAD model. We acquired MRI data in two cohorts: 12 mice in the first cohort and 11 mice in the second cohort. We excluded one female mouse from 5xFAD;*APOE3* group due to the severe motion and noise artifacts. Therefore, we analyzed MRI data from 11 mice/genotype. We present MRI data from 11 5xFAD;*APOE3* (9 males, 2 females) and 11 5xFAD;*APOE4* (7 males, 4 females) mice. The animals were immediately sacrificed after the MRI scan. The brain tissues were processed subsequently for histological analysis.

### 2.3 T_2_‐weighted data processing and voxel‐based morphometry analysis

T_2_‐weighted images were denoised^35^ and bias corrected^36^ prior to removing the skull for voxel‐based analysis. To identify morphological alterations at the voxel level, we performed a voxel‐based analysis via a well‐established method.^37^ An average T_2_‐weighted structural image was created via a series of affine and diffeomorphic registration protocols available in Advanced Normalization Tools (ANTs).^36^ We subsequently calculated the Jacobian determinant map for each sample via the diffeomorphic transformation matrices. The map contains information on local volumetric changes (shrinkage and expansion) relative to the template. Voxel‐wise *t*‐test (5xFAD;*APOE3* vs. 5xFAD;*APOE4*) were performed to detect local volumetric structural changes via “randomize” (FSL),^38^ with a design matrix of (11, 11). We performed 5000 permutations and applied family‐wise error correction.^39^

### 2.4 Diffusion and NODDI data processing

We preprocessed the diffusion MRI data via open access software. Specifically, we corrected head motion and eddy current correction via FSL,^40^ and corrected biasfield via ANTs.^36^ The bias‐corrected diffusion data were used to manually create the mask for skull extraction. Preprocessed DWI data with *b*‐values = 600 and 1200 s/mm^2^ were used to generate scalar images, such as fractional anisotropy, axial diffusivity, and radial diffusivity, via MRtrix3.^41^ To compute NODDI values, we used multishell preprocessed DWI data. Briefly, multishell *b* values of 0, 600, 1200, 1800, and 2400 s/mm^2^ were fitted via the NODDI toolbox (MATLAB)^42^ with a diffusion parameter (*d_par _* = 0.001). The NODDI metrics, including the neurite density index (NDI), orientation dispersion index (ODI), and isotropic volume fraction (ISO), were generated via Watson's turtosity model, where neurites were considered impermeable sticks (cylinders with zero radius) in a homogeneous background. We registered images such as fractional anisotropy, axial diffusivity, radial diffusivity, the NDI, the ODI, and the ISO to the Australian Mouse Brain Mapping Consortium (AMBMC) atlas^43^ via ANTS.^36^

### 2.5 Segmentation and determination of scalar parameters from diffusion MRI data

To generate seed‐based tractography, we manually created seeds for three white matter tracts (anterior commissure, hippocampal commissure, and corpus callosum) on color fractional anisotropy maps in ITK‐SNAP.^44^ Probabilistic tractography was derived from the regions of interest, employing 50 seeds per voxel. The resulting tractograms were subsequently transformed into track‐density maps, and a 10% threshold was applied to eliminate spurious signals from the probabilistic tractography. Tractogram maps were then utilized to measure the scalar parameters within each of the specified white matter structures.

### 2.6 Tractography

The whole‐brain structural connectome was generated via a previously established method.^37^ In essence, a whole‐brain probabilistic tractography was generated with 10 seeds per voxel. We employed a combination of AMBMC and John Hopkins C57BL/6J mouse brain atlases^37^ to register the *b* = 0 image of the individual brain. The “tck2connectome” command (MRtrix3) was used to generate the connectivity matrix from 76 × 76 brain regions (Table S1).

### 2.7 Structural connectivity analysis

To identify vulnerable connections and ascertain the global differences in structural connectivity between 5xFAD;*APOE3* and 5xFAD;*APOE4* mouse brains, we performed network‐based statistical analysis.^45^ Weighted connections were incorporated into the network‐based statistical analysis. We performed 5000 permutations within a threshold range of 2.5–3.5. We used two opposite contrasts (5xFAD;*APOE3 *> 5xFAD;*APOE4* and 5xFAD;*APOE3 *< 5xFAD;*APOE4*) in structural connectivity analysis.

### 2.8 Analysis of the network

To investigate network properties, we applied graph theory. Given the probability of false connections arising from probabilistic tractography, we employed a reduced sparsity network (30%). Key graph theory parameters, including betweenness centrality, degree centrality, global efficiency, assortativity, local efficiency, clustering coefficient, shortest path length, and small‐worldness, were calculated via the GRETNA toolbox (MATLAB).^46^

### 2.9 Tissue collection

The mice were anesthetized with Avertin (250 mg/kg, intraperitoneally) and perfused transcardially with ice‐cold phosphate‐buffered saline. The brain was immediately dissected, and the left hemisphere was fixed in 4% paraformaldehyde combined with 0.01 M lysine and 0.01 M sodium metaperiodate for 24 h at 4°C. The brain sections were cryoprotected in 30% sucrose and embedded in optimal cutting temperature compound (OCT). Coronal brain sections (20 µm thick) were cut via a Leica cryostat and mounted on slides.

### 2.10 Luxol fast blue staining

To assess myelination in the white matter, Luxol Fast Blue (Fisher Scientific, Cat# AC212170250) staining was conducted. The sections were incubated in Luxol fast blue (LFB) solution for 1 h at 60°C, followed by an overnight incubation at 40°C. Lithium carbonate was used to differentiate the sections until clear differentiation was observed between the gray matter and white matter.

### 2.11 Immunostaining

Antigen retrieval was performed via antigen retrieval solution (Invitrogen, Cat# 00‐4956‐58) at 90°C for 20 min. The sections were then blocked with a solution containing 5% normal goat serum and 0.1% Triton X‐100 for 1 h, followed by an overnight incubation with primary antibodies. We used primary antibodies against oligodendrocyte transcription factor 2 (OLIG2) (Millipore, Cat#AB9610), myelin basic protein (MBP) (EnCor Biotechnology Inc, Cat#P02687), adenomatous polyposis coli (APC or “CC1” clone) (Millipore, Cat#OP80), brain enriched myelin associated protein 1 (BCAS1) (Synaptic systems, Cat#445004), and ionized calcium binding adapter molecule 1 (IBA1) (Wako laboratories, Cat#019‐19741). The sections were then washed and further incubated with Alexa Fluor 488/555/647 secondary antibodies for 2 h before being mounted in an aqueous medium. To quantify the accumulation of amyloid plaque load, we used 1,4‐bis(3‐carboxy‐4‐hydroxyphenylethenyl)benzene, also known as X‐34 (Fisher Scientific), which detects fibrillar plaques. The 4′,6‐diamidino‐2‐phenylindole (DAPI) was used for nuclear staining. In case of histological analysis, we used sections from the second MRI cohort. In the first MRI cohort, we immunostained six mice per genotype with the paraformaldehyde fixation method. Unfortunately, BCAS1 immunostaining failed in the first MRI cohort. The fixation conditions with paraformaldehyde alone were insufficient for preserving the antigenicity of BCAS1. Therefore, we had to change our fixation method to paraformaldehyde combined with lysine and sodium metaperiodate^47^ with our second MRI cohort, consisting of six 5xFAD;*APOE3* and five 5xFAD;*APOE4* mice. All mice from the second cohort were immunostained.

### 2.12 Analysis of stained brain sections

To assess myelination, we acquired brightfield imaging data from LFB‐stained brain sections and quantified the intensity of LFB. The intensity of LFB staining was calculated via Fiji.^48^ To quantify the MBP intensity, CC1‐, IBA1‐, and BCAS1‐positive cells, and OLIG2‐positive nuclei, we acquired fluorescence images using an inverted fluorescence microscope (Leica Thunder Biosystems). The average of three to six sections (starting 2.00–2.80 mm posterior to bregma) from different anatomical coordinates (150 µm distance) was calculated. We calculated the percentage of the area covered by MBP using Fiji software. To count CC1‐, BCAS1‐, and IBA1‐positive cells and OLIG2‐positive nuclei, we first classified signals and noise via the machine learning technique available in Ilastik v1.3.3.^49^ The probability maps of the signals were imported into CellProfiler v4.2.5.^50^ We then used in‐house‐developed modules in CellProfiler to count colocalized OLIG2‐ and CC1‐positive mature oligodendrocytes and OLIG2‐ and BCAS1‐positive actively myelinating oligodendrocytes. The number of cells was normalized to the total area analyzed. X‐34‐stained images were directly imported to Fiji to quantify the percentage area of X‐34 staining.

### 2.13 Statistical analysis

GraphPad PRISM (version 10.4.2) was used to perform statistical analyses. We performed an unpaired *t*‐test to compare 5xFAD;*APOE3* and 5xFAD;*APOE4* mice. Voxel‐based morphometric analysis via a *t*‐test followed by multiple comparison correction was also conducted to evaluate the differences between 5xFAD;*APOE3* and 5xFAD;*APOE4* mice.Network‐based statistics analysis was used to identify vulnerable connections between 5xFAD;*APOE3* and 5xFAD;*APOE4* mice. *p*‐values less than 0.05 were considered statistically significant.

## 3 RESULTS

### 3.1 MRI analysis revealed microstructural abnormalities in 5xFAD;*APOE4* mice

To determine the effects of *APOE* isoforms on neuronal integrity, we first calculated scalar values from magnetic resonance images by registering an AMBMC atlas to the structural (T_2_‐weighted), diffusion tensor (fractional anisotropy, axial diffusivity, radial diffusivity), and neurite density and dispersion images (NDI, ODI, isotropic volume fraction).

**FIGURE 1:**
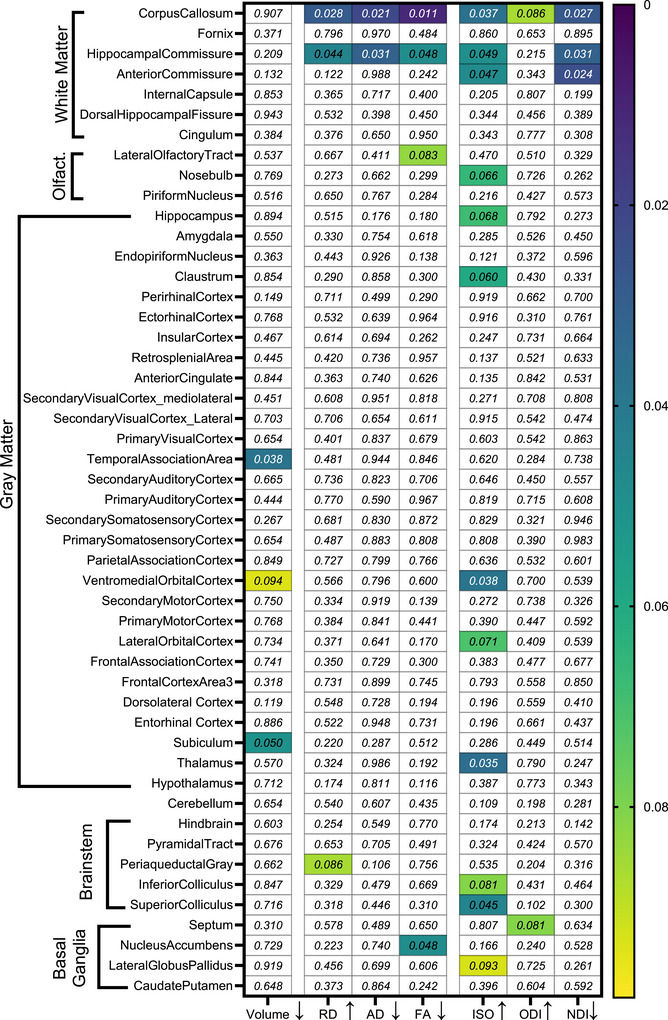
Region‐specific changes in MRI metrics. The heatmap shows *p*‐values derived from unpaired *t‐*tests comparing 5xFAD;*APOE3* and 5xFAD;*APOE4* mice from the atlas‐based analysis. The horizontal axis shows the assessed metrics, including volume difference, RD, AD, FA, ISO, ODI, and NDI. The gradient color bar represents the *p*‐value strength from 0.000 (deep blue) to 0.090 (yellow). The vertical axis denotes the analyzed brain structures and regions. The arrow indicates the direction of change, ↑, increase; ↓, decrease. *n* = 11/genotype. AD, axial diffusivity; APOE, apolipoprotein E; FA, fractional anisotropy; ISO, isotropic volume fraction; MRI, magnetic resonance imaging; NDI, neurite density index; ODI, orientation dispersion index; RD, radial diffusivity.

We observed altered MRI metrics in several brain structures between 5xFAD;*APOE3* and 5xFAD;*APOE4* mice (Figure 1). Both the diffusion and NODDI metrics indicated region‐specific changes in 5xFAD;*APOE4* mice. 5xFAD;*APOE4* mice had reduced structural atrophy in the temporal association area and subiculum (Figure 1, “Volume” column), alongside increased radial diffusivity in the corpus callosum and hippocampal commissure (Figure 1, “RD” column). Additionally, decreases in axial diffusivity (Figure 1, “AD” column), fractional anisotropy (Figure 1, “FA” column), and NDI (Figure 1, “NDI” column) were detected in these same white matter tracts of 5xFAD;*APOE4* mice (Figure 1). Importantly, multiple brain regions exhibited increased isotropic volume fraction (Figure 1, “ISO” column), without affecting ODI (Figure 1, “ODI” column), demonstrating that ISO may serve as a biomarker sensitive to *APOE*‐isoform‐specific effects, potentially reflecting underlying microstructural alterations. Among all regions analyzed, the corpus callosum demonstrated the most pronounced alterations across all MRI metrics (Figure 1, topmost row), highlighting its vulnerability to *APOE4* genotype‐associated structural changes.

To confirm this structural atrophy observed in registration‐based analysis (Figure 1, “Volume” column), we performed voxel‐based morphometry analysis on T_2_‐weighted images (Figure S1A, B). We first created a study‐specific template comprising all 23 samples. Voxel‐based morphometry analysis revealed a volume reduction in the striatum, external capsule, insular cortex, hippocampal commissure, corpus callosum, inferior colliculus, entorhinal cortex, and periaqueductal gray, indicating atrophy of these brain regions in 5xFAD;*APOE4* mice compared to 5xFAD;*APOE3* mice (Figure S1A, B). Statistically significant volume reduction is shown in the colored voxel in Figure S1A, and a 3D view is shown in Figure S1B. The discrepancy between the two approaches (atlas‐based registration vs. voxel‐based morphometry) was not unexpected. We used a pathological mouse model, that is, 5xFAD, to register with a healthy mouse (AMBMC atlas).^43^ Therefore, the delineation of the structural boundaries during the registration process can raise this difference. However, voxel‐based morphometry enables whole‐brain analysis of structural differences without predefined regions, allowing the detection of subtle anatomical variations between groups.

### 3.2 Tract‐specific disruption of white matter integrity in 5xFAD;*APOE4* mice

**FIGURE 2:**
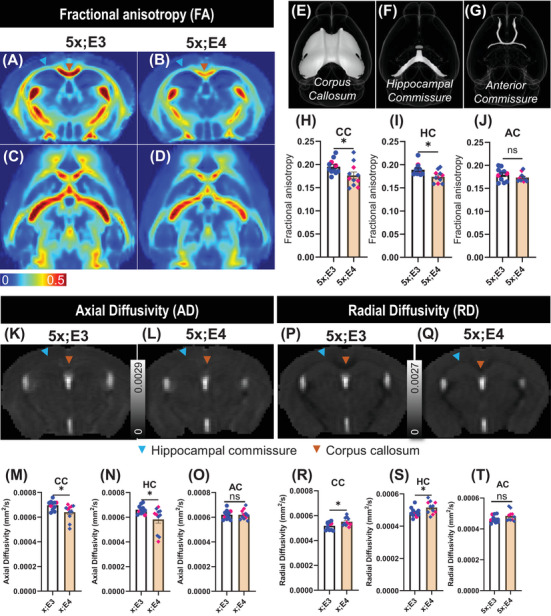
Tract‐specific analysis identifies altered diffusion MRI metrics in 5xFAD;*ApoE4* mice. (A, B) Coronal and (C, D) axial images depicting mean FA for each genotype. Scale bar: 0–0.5. White matter tracts analyzed include the CC (E), HC (F), and AC (G). (H, I, J) Tract‐specific FA values for each white matter tract. (K, L) Mean AD image of each genotype. Scale bar: 0–0.0029. (M, N, O) Tract‐specific axial diffusivity value for each white matter tract. (P, Q) Mean radial diffusivity image of each genotype. Scale bar: 0–0.0027. (R, S, T) Tract‐specific radial diffusivity values for CC, HC, and AC brain regions. An unpaired *t*‐test was performed between 5x;E3 (5xFAD;*APOE3*) and 5x;E4 (5xFAD;*APOE4*) groups. Data are presented as mean ± SEM, with blue and pink bullets representing male and female mice, respectively, with *n* = 11/genotype. AC, anterior commissure; AD, axial diffusivity; APOE, apolipoprotein E; CC, corpus callosum; FA, fractional anisotropy; HC, hippocampal commissure; MRI, magnetic resonance imaging; RD, radial diffusivity; SEM, standard error of the mean. *p < 0.05 and ns = not significant.

To visualize the group‐level representation of fractional anisotropy images, we prepared mean fractional anisotropy images for each genotype. These mean images provide a reference for assessing white matter integrity across the brain. We found *APOE4* reduced fractional anisotropy in the corpus callosum (Figure 2A–D, orange arrowhead, Figure S2Aa) and hippocampal commissure (Figure 2A–D, blue arrowhead, Figure S2Ab) regions. Based on these observations, we selected three major white matter tracts, the corpus callosum (Figure 2E), hippocampal commissure (Figure 2F), and the anterior commissure (Figure 2G), to conduct seed‐based tractography analyses. These tracts were chosen because atlas‐based analysis and mean fractional anisotropy images indicated particularly compromised integrity in these regions.

While atlas‐based analysis provides a general overview of neuronal integrity, it does not reveal tract‐specific microstructural properties of white matter. To address this limitation, we performed analyses of the tract‐specific properties within these white matters. Compared with 5xFAD;*APOE3* mice, 5xFAD;*APOE4* mice had a significant reduction in fractional anisotropy within the corpus callosum [*t*_20_ = 2.82, *p* = 0.010] (Figure 2H) and hippocampal commissure [*t*_20_ = 2.17, *p* = 0.048] (Figure 2I) but not in the anterior commissure [*t*_20_ = 1.20, *p* = 0.242] (Figure 2J). Because the values of fractional anisotropy reflect the integrity and organization of axons as well as the extent of myelination,^51^ our data suggest that *APOE4* exacerbates white matter vulnerabilities in AD. Consistent with the fractional anisotropy findings, the mean axial diffusivity image also revealed reduced values in the corpus callosum and hippocampal commissure of 5xFAD;*APOE4* mice (Figure 2K, L, Figure S2Ba,b). Because axial diffusivity reflects axonal integrity, the decreased values in 5xFAD;*APOE4* mice suggest axonal injury or degeneration.^52^ Additionally, tract‐based analysis revealed a significant reduction in axial diffusivity within the corpus callosum [*t*_20_ = 2.50, *p* = 0.020] (Figure 2M) and the hippocampal commissure [*t*_20_ = 2.42, *p* = 0.024] (Figure 2N) of 5xFAD;*APOE4* mice, whereas the anterior commissure remained unaffected [*t*_20_ = 0.01, *p* = 0.988] (Figure 2O). In contrast to the axial diffusivity data, higher radial diffusivity values were found in the corpus callosum and hippocampal commissure regions of 5xFAD;*APOE4* mice within the mean radial diffusivity image (Figure 2P, Q, Figure S2Ca,b). Because radial diffusivity reflects a magnitude of water diffusion perpendicular to axonal fibers, our data suggest that 5xFAD;*APOE4* mice had myelin loss.^52^ Tract‐based analysis further revealed significantly higher radial diffusivity in the corpus callosum [*t*_20_ = 2.36, *p* = 0.028] (Figure 2R) and hippocampal commissure [*t*_20_ = 2.14, *p* = 0.045] (Figure 2S) of 5xFAD;*APOE4* mice but not in the anterior commissure [*t*_20_ = 1.61, *p* = 0.122] (Figure 2T). Taken together, these reductions in fractional anisotropy and axial diffusivity, coupled with increased radial diffusivity, indicate *APOE4* disrupted fiber tracts and demyelination selectively in the corpus callosum and hippocampal commissure.

### 3.3 *APOE4* allele impairs neurite density and increases the free water content in white matter

While diffusion MRI provides valuable information about white matter structure, it does not provide estimates of neurite density, fanning of neurites, or partial volume of cerebrospinal fluid. To overcome these limitations, we applied an innovative NODDI approach to examine neurite and dendritic density as well as the partial volume of cerebrospinal fluid. The signal‐to‐noise ratio was maximized using a cryogenically cooled radio frequency coil in a 9.4T MRI scanner. Cryoprobe enables more precise identification of subtle anatomical and microstructural alterations. To calculate NODDI metrics, we used an algorithm that estimates hindered diffusivity, free diffusivity, and neurite packing density by applying Watson's tortuosity model for randomly packed cylinders.^42^ The NDI, ODI, and ISO were calculated using this algorithm.

**FIGURE 3:**
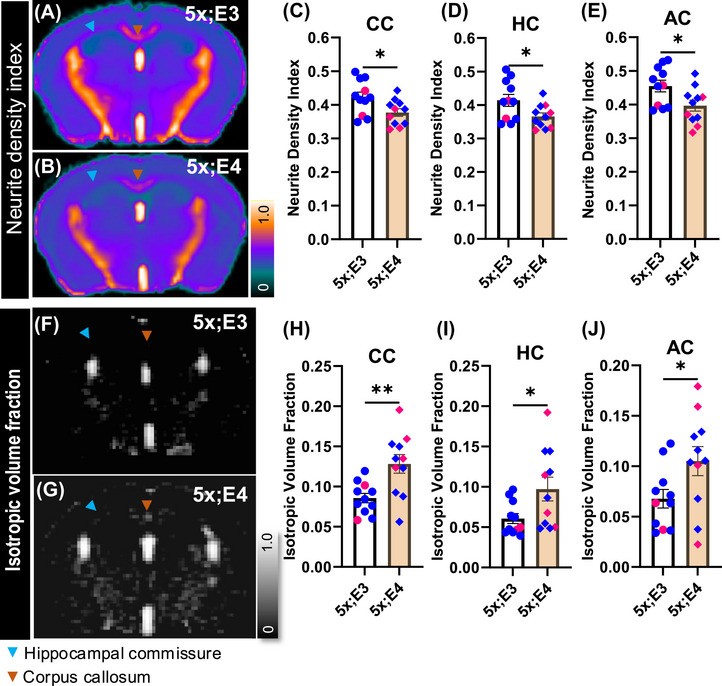
Tract‐specific analysis identifies altered NODDI MRI metrics in 5xFAD;*APOE4* mice. (A, B) Mean NDI image for each genotype. Scale bar: 0–1. (C, D, E) Tract‐specific NDI values for CC, HC, and AC. (F, G) Mean ISO image for each genotype. Scale bar: 0–1. (H, I, J) Tract‐specific ISO values for CC, HC, and AC. Unpaired *t*‐test was performed between the groups 5x;E3 (5xFAD;*APOE3*) and 5x;E4 (5xFAD;*APOE4*). Data are presented as mean ± SEM, with blue and pink bullets representing male and female mice, respectively, with *n* = 11/genotype. AC, anterior commissure; APOE, apolipoprotein E; CC, corpus callosum; HC, hippocampal commissure; ISO, isotropic volume fraction; MRI, magnetic resonance imaging; NDI, neurite density index; NODDI, neurite orientation dispersion and density imaging; SEM, standard error of the mean. *p < 0.05 and **p < 0.01.

The NDI reflects the proportion of the intraneurite space. We first prepared a mean NDI image and observed a lower signal in the corpus callosum and hippocampal commissure of 5xFAD;*APOE4* mice (Figure 3A, B, Figure S2Da,b). We subsequently extracted tract‐specific NDI values and detected a significant reduction in the NDI in 5xFAD;*APOE4* mice compared with 5xFAD;*APOE3* mice across three major white matter tracts, corpus callosum [*t*_20_ = 2.38, *p* = 0.028] (Figure 3C), hippocampal commissure [*t*_20_ = 2.31, *p* = 0.034] (Figure 3D), and anterior commissure [*t*_20_ = 2.44, *p* = 0.024] (Figure 3E). These data demonstrate that *APOE4* decreased the density of neurites in the corpus callosum, hippocampal commissure, and anterior commissure, demonstrating less densely packed neurites in those tracts.

The ODI indicates how coherently or randomly neurites are aligned in the tissue microstructure. A low ODI indicates that neurites are highly aligned and oriented in a similar direction, as observed in regions such as the white matter tracts. Our mean ODI maps (Figure S2Ea,b) did not show any noticeable differences in the corpus callosum (Figure S2Ec), hippocampal commissure (Figure S2Ed), and anterior commissure (Figure S2Ee). Despite this reduction in neurite density, the ODI remained unchanged across these tracts, indicating that the remaining neurites retain their coherent alignment and directional organization typical of healthy white matter. This result demonstrates that while *APOE4* promotes axonal or dendritic loss, it does not disrupt the overall structural orientation of the surviving fibers, potentially preserving some aspects of tract function even as cellular density declines.

We next prepared average ISO images for each genotype and calculated ISO values between the genotypes (Figure 3F, G; Figure S2Fa,c). ISO represents the proportion of water in a voxel that diffuses isotropically. We found a significantly greater ISO value in corpus callosum [*t*_20_ = 3.26, *p* = 0.003] (Figure 3H), hippocampal commissure [*t*_20_ = 2.28, *p* = 0.034] (Figure 3I), and anterior commissure [*t*_20_ = 2.18, *p* = 0.040] (Figure 3J) of 5xFAD;*APOE4* mice, reflecting higher free water content in the 5xFAD;*APOE4* mice than in the 5xFAD;*APOE3* mice. Collectively, these findings demonstrate that *APOE4* impairs various parameters associated with white matter integrity in 5XFAD mice.

### 3.4 *APOE4*‐driven disruption of the structural network

**FIGURE 4:**
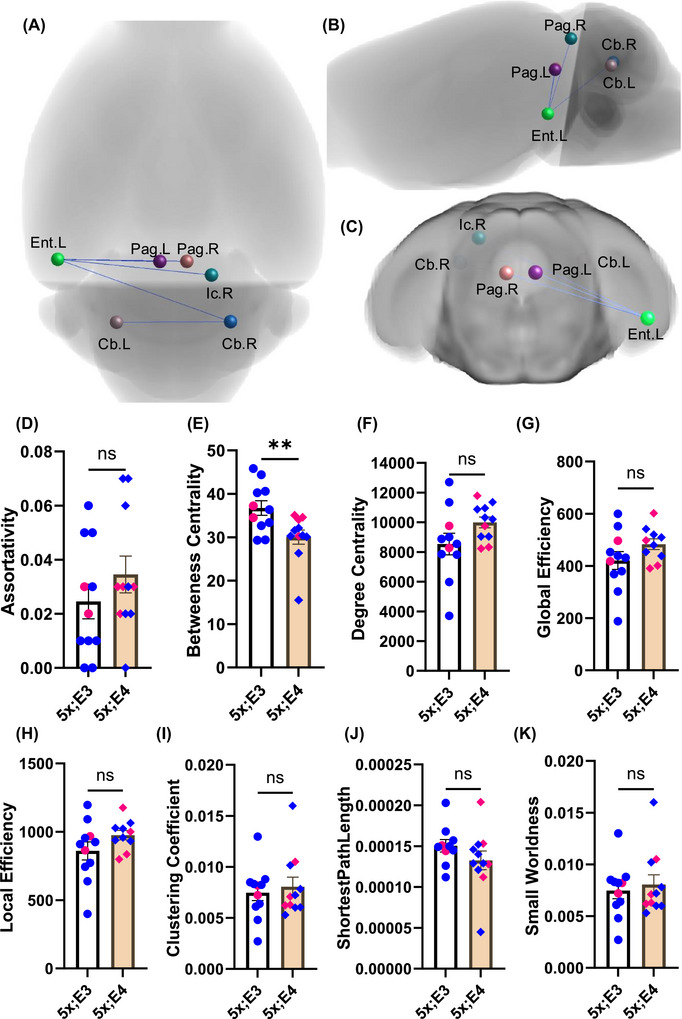
Disrupted structural connectivity in 5xFAD;*APOE4* mice. (A) Axial, (B) sagittal and (C) coronal views of disrupted structural connectivity in 5xFAD;*APOE4* mice analyzed by the network‐based statistical approach. Ent. L (left entorhinal cortex), Cb. L (left cerebellum), Cb. R (right cerebellum), Pag. L (left periaqueductal gray), Pag. R (right periaqueductal gray), and Ic. R (right inferior colliculus). This disrupted network was identified at a statistical threshold of 3.0 with the contrast 5xFAD;*APOE3* > 5xFAD;*APOE4*. The networks presented are significant at *p*  <  0.05, FDR corrected. (D–K) Network‐wise comparisons between 5xFAD;*APOE3* and 5xFAD;*APOE4* mice. Network parameters include (D) assortativity, (E) betweenness centrality, (F) degree centrality, (G) global efficiency, (H) local efficiency, (I) clustering coefficient, (J) shortest path length, and (K) small worldness. Data are presented as mean ± SEM, with blue and pink bullets representing male and female mice, respectively, with *n* = 11/genotype. APOE, apolipoprotein E; FDR, false discovery rate; SEM, standard error of the mean. **p < 0.01 and ns = not significant.

To investigate the impact of disrupted integrity on structural connectivity, we conducted a network‐based statistics analysis. To resolve crossing fiber ambiguities in tractography, we applied a multishell diffusion MRI approach with five distinct *b* values. Our protocol, combining 116 directions across shells, significantly improved angular resolution for whole brain structural connectivity mapping. Our protocol reduced false‐positive tracking errors compared to single shell approaches. The network‐based statistics analysis revealed disrupted structural connectivity in 5xFAD;*APOE4* mice at a statistical threshold of *t *= 3.0, with the contrast 5xFAD;*APOE3 *> 5xFAD;*APOE4* (Figure 4A–C). In the disrupted network, the nodes were the left entorhinal cortex, right insular cortex, right and left periaqueductal gray, and right and left cerebellum. The left entorhinal cortex had the highest number of connectivity disruptions (Figure 4A, B).

While network‐based statistics identify specific connections or subnetworks with altered connectivity, graph theoretical network analysis complements this by revealing how these changes impact the global and local organizational properties of the brain, providing a comprehensive understanding of the underlying disruptions in neuronal communication and network efficiency. Graph network metrics, such as assortativity (Figure 4D), degree centrality (Figure 4F), global efficiency (Figure 4G), local efficiency (Figure 4H), the clustering coefficient (Figure 4I), shortest path length (Figure 4J), and small‐worldness (Figure 4K), did not significantly differ between the two groups. However, we detected a significant reduction in betweenness centrality [*t*_20_ = 2.85, *p* = 0.009] in 5xFAD;*APOE4* mice (Figure 4E). The reduced betweenness centrality in 5xFAD;*APOE4* mice demonstrates a diminished role of certain brain regions as critical hubs for information flow, potentially reflecting impaired integration and disrupted communication pathways within the neuronal network.

### 3.5 *APOE4* decreases the level of myelination in 5xFAD mice

**FIGURE 5:**
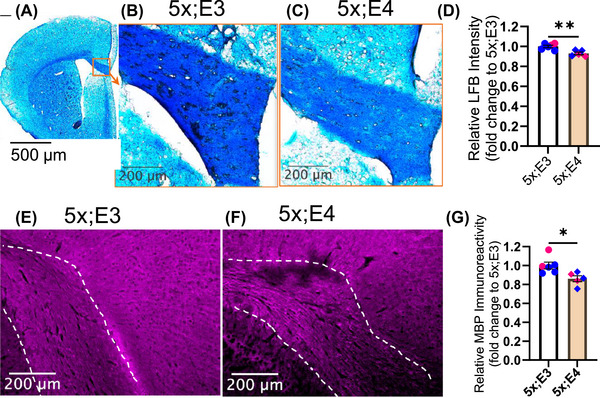
Reduced myelin in 5xFAD;*APOE4* mice. (A) Representative images of LFB‐stained brain section. Scale bar: 500 µm. (B, C) Representative LFB‐stained images of the corpus callosum for each genotype. Scale bar: 200 µm. (D) Relative LFB intensity (fold change to 5xFAD;*APOE3*) in the CC. (E, F) Representative images of MBP‐immunostained sections for each genotype. Scale bar: 200 µm. (G) Relative MBP immunoreactivity (fold change to 5xFAD;*APOE3*) in the corpus callosum. Unpaired *t* test between 5x;E3 (5xFAD;*APOE3*) (*n* = 6) and 5x;E4 (5xFAD;*APOE4*) (*n* = 5) mice. Data are presented as mean ± SEM, blue and pink bullets represent male and female mice, respectively. APOE, apolipoprotein E; CC, corpus callosum; LFB, Luxol fast blue; MBP, myelin basic protein; SEM, standard error of the mean. *p < 0.05 and **p < 0.01.

Given that both diffusion (Figure 2) and NODDI (Figure 3) MRI data indicate microstructural deficits, we further investigated the extent of myelination. To examine the neuroanatomical underpinnings of myelin deficits, we stained brain sections with LFB. Because LFB binds to lipids found in myelin, it was used to selectively stain the myelinated axons (Figure 5A–C). We detected a significant reduction in LFB intensity [*t*_9_ = 3.85, *p* = 0.003] in the corpus callosum of 5xFAD;*APOE4* mice compared with 5xFAD;*APOE3* mice (Figure 5D). To further corroborate this finding, we performed immunostaining for MBP, a key structural component of the myelin sheath, providing a cellular‐level assessment to determine whether reduced myelin content underlies these microstructural alterations (Figure 5E, F). Consistent with the LFB results, we observed a significant decrease in the MBP‐positive area [*t*_9_ = 2.86, *p* = 0.018] in the corpus callosum of 5xFAD;*APOE4* mice (Figure 5G). These results collectively indicate decreased levels of myelination, particularly in the corpus callosum in 5xFAD;*APOE4* mice. Our data demonstrate that APOE4 protein may cause demyelination or impaired myelin maintenance.

### 3.6 *APOE4* decreases the number of mature and actively myelinating oligodendrocytes, but not the overall oligodendrocyte lineage cell population *in 5xFAD mice*

**FIGURE 6:**
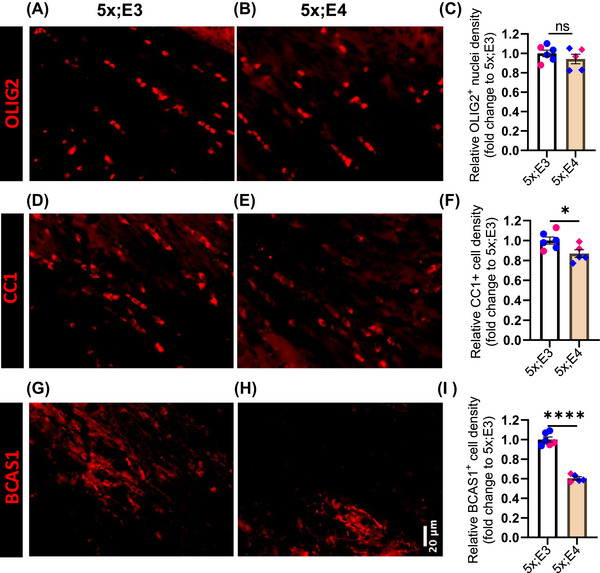
Reduced number of mature and actively myelinating oligodendrocytes in 5xFAD;*APOE4* mice. (A, B) Representative OLIG2‐immunostained images of oligodendrocyte lineage cells in the CC for each genotype. (C) Relative OLIG2‐positive nuclei density (fold change to 5xFAD;*APOE3*) in the corpus callosum. (D, E) Representative CC1‐immunostained images of mature oligodendrocytes in the corpus callosum for each genotype. (F) Relative CC1‐positive cell density (fold change to 5xFAD;*APOE3*) in the corpus callosum. (G, H) Representative BCAS1‐immunostained images of actively myelinating oligodendrocytes in the CC for each genotype. (I) Relative BCAS1‐positive cell density (fold change to 5xFAD;*APOE3*) in the corpus callosum. Data are presented as mean ± SEM. Statistical comparisons were performed using unpaired *t‐*tests between 5xFAD;*APOE3* (*n* = 6) and 5xFAD;*APOE4* (*n* = 5) mice. Scale bar: 20 µm. Blue and pink markers represent male and female mice, respectively. APOE, apolipoprotein E; BCAS1, brain enriched myelin associated protein 1; CC, corpus callosum; OLIG2, oligodendrocyte transcription factor 2; SEM, standard error of the mean. *p < 0.05, ****p < 0.0001 and ns = not significant.

To further investigate the cellular mechanism of myelin deficiency (Figure 5), we analyzed the different types of oligodendrocytes. First, to detect all oligodendrocyte lineage cells, we stained brain sections with anti‐OLIG2 antibody (Figure 6A, B) and counted anti‐OLIG2 antibody‐positive nuclei (Figure 6C). There was no significant difference in the number of oligodendrocyte lineage cells [*t*_9_ = 1.01, *p* = 0.340] between the two genotypes (Figure 6C). Next, to identify mature oligodendrocytes, we stained brain sections with CC1 antibody (Figure 6D, E) that binds to an RNA‐binding protein, Quaking 7,^53^ which is highly expressed in mature oligodendrocytes (Figure 6D, E), and counted CC1 antibody‐positive cells (Figure 6F). We observed a significant decrease in CC1‐positive mature oligodendrocytes in 5xFAD;*APOE4* mice compared with those in 5xFAD;*APOE3* mice [*t*_9_ = 2.50, *p* = 0.033] (Figure 6F). These data suggest that there is a selective loss of mature oligodendrocytes, although there was no global dysregulation of the total oligodendrocyte lineage cell population. Given the selective loss of mature oligodendrocytes, we next assessed whether active myelination processes were also compromised in these mice. To identify actively myelinating oligodendrocytes, we performed immunostaining of brain sections with an anti‐BCAS1 antibody (Figure 6G, H). The number of anti‐BCAS1 antibody‐positive cells in 5xFAD;*APOE4* mice was significantly lower [*t*_9_ = 12.12, *p* = 0.0001] than that of 5xFAD;*APOE3* mice (Figure 6I). This substantial reduction in the number of BCAS1‐positive cells suggests a marked decrease in active myelination in the 5xFAD;*APOE4* mice. Taken together, our findings demonstrate that *APOE4* contributes to reduced numbers of mature (Figure 6F) and actively myelinating (Figure 6I) oligodendrocytes in the white matter of 5xFAD mice. These reductions are likely to be underlying mechanisms for the observed impairment in white matter integrity (Figure 2) and structural connectivity (Figure 4A, C).

### 3.7 *APOE4* increases plaque deposition and gliosis

To elucidate the possible factors contributing to myelin deficiency, we analyzed plaque deposition using X‐34 dye that detects β‐sheet structure proteins (Figure S3A, B). Our analysis revealed a marked increase in X‐34‐positive fibrillar plaques in the corpus callosum in 5xFAD;*APOE4* mice (Figure S3C). Although sex differences in amyloid plaque deposition have been reported in the hippocampus and cortex of 5xFAD mice, we found no sex difference in amyloid levels in the corpus callosum (Figure S3D). Given the established link between amyloid pathology and neuroinflammatory responses,^54^, ^55^ we then quantified changes in microglia in the corpus callosum using an IBA1 antibody (Figure S3E, F). Concomitant with the observed increase in plaque deposition, we detected significantly increased microgliosis in 5xFAD;*APOE4* mice (Figure S3G). These findings suggest that *APOE4* exacerbates amyloid‐β deposition in myelinated regions, such as the corpus callosum, which in turn may trigger pronounced microglial activation. This amyloid‐driven neuroinflammation may disrupt myelin integrity, accelerating white matter impairment in 5xFAD mice.

### 3.8 Correlation analyses between MRI metrics and histological markers

To elucidate the cellular basis of white matter impairment observed in our MRI experiment, we examine the relationship between MRI‐derived metrics and histological markers (Figure S4). Notably, MBP immunostaining, a marker of myelination, exhibited a significant positive correlation with fractional anisotropy (*p* = 0.047) (Figure S4A). This finding suggests that higher fractional anisotropy is indicative of greater coherence of white matter tracts, consistent with preserved or increased myelin content. Conversely, MBP immunostaining showed a negative correlation with both radial diffusivity (*p* = 0.011) (Figure S4B) and isotropic volume fraction (*p* = 0.035) (Figure S4C). Higher radial diffusivity and isotropic volume fraction have been associated with increased water content and reduced myelin integrity.

We further investigated the association between amyloid pathology and MRI measures. Amyloid plaque deposition exhibited a significant negative correlation with NDI (*p* = 0.042) (Figure S4D) and was positively correlated with isotropic volume fraction (*p* = 0.001) (Figure S4E). This result suggests that amyloid accumulation is linked to microstructural alterations and changes in tissue microenvironment. Finally, We observed that IBA1 immunostaining, a marker of myeloid cell activation and neuroinflammation, was positively correlated with radial diffusivity (*p* = 0.001) (Figure S4F). This relationship implies that the neuroinflammatory process contributes to increased water diffusion perpendicular to axonal fibers.

## 4 DISCUSSION

The *APOE4* allele is a well‐established risk factor for AD. Previous studies have investigated the impact of *APOE* isoforms on white matter integrity in AD patients.^11^, ^22^, ^56^ However, preclinical research with animal models is necessary to elucidate the cellular mechanisms by which *APOE* isoforms influence white matter structure. To address this critical gap, we established an Aβ‐amyloidosis mouse model expressing either *APOE3* or *APOE4*, recapitulating the *APOE4*‐associated white matter phenotype observed in clinical studies while enabling mechanistic investigation of *APOE4*‐related white matter impairment. Using the advanced MRI technique (NODDI), we assessed white matter integrity in 5xFAD;*APOE3* and 5xFAD;*APOE4* mice. Our results demonstrated that, compared with 5xFAD;*APOE3*, 5xFAD;*APOE4* mice had significant reductions in fractional anisotropy (Figure 2H, I) and neurite density indices (Figure 3C, E) within major white matter tracts, along with a marked disruption of structural connectivity (Figure 4A, C). Our study identified key NODDI‐derived metrics that can serve as a novel in vivo longitudinal platform for evaluating therapeutic interventions.

To further investigate the cellular mechanism underlying these white matter impairments observed in the MRI analyses, we analyzed multiple types of oligodendrocyte lineage cells in brain sections from the same cohort of mice used for MRI scans. We observed reduced myelination (Figure 5G) in 5xFAD;*APOE4* mice, likely due to a decreased number of actively myelinating mature oligodendrocytes (Figure 6I). Therefore, our data suggest that *APOE4* compromises the integrity of white matter by reducing the population of actively myelinating oligodendrocytes. These results provide new insight into the mechanisms underlying the increased susceptibility to white matter damage in *APOE4* carriers and may help explain the higher prevalence of white matter hyperintensities observed in this population. Restoring the population of actively myelinating oligodendrocytes may provide a novel therapeutic strategy for AD.

### 4.1 Biological implications of altered diffusion MRI metrics

In healthy white matter, water diffuses primarily in the direction of myelinated axons. Demyelination or axonal damage reduces diffusion restriction, decreasing fractional anisotropy. 5xFAD;*APOE4* mice exhibit lower fractional anisotropy (Figure 2H, I), demonstrating disrupted organization and demyelination in white matter tracts.^51^ Mechanistically, myelin loss may loosen axonal sheaths, enhancing perpendicular diffusion of water, leading to^52^ lower fractional anisotropy.

### 4.2 Biological interpretation of altered NODDI MRI metrics

To identify the causes of reduced fractional anisotropy within white matter (Figure 2H, I), we used NODDI to assess intraneurite, extraneurite, and isotropic changes. Loss of axons and dendrites decreases the intraneurite space, as reflected by a decrease in the NDI. This reduction is often accompanied by an expansion of the extraneurite space, indicated by a higher ODI, or by an accumulation of cerebrospinal fluid, indicated by a higher isotropic volume fraction. A *post mortem* AD study in humans revealed a decrease in NDI in the white matter of *APOE4* carriers, suggesting a loss of axons.^56^ Consistent with these findings in humans, we also observed a reduction in NDI in the *APOE4* mouse model of Aβ amyloidosis (Figure 3C, E), indicating axonal loss. Notably, 5xFAD;*APOE4* mice had higher isotropic volume fraction than 5xFAD; *APOE3* mice (Figure 3H, J), suggesting increased accumulation of water in less myelinated white matter. These findings collectively indicate that *APOE4* induces significant microstructural changes in white matter, including reduced neurite density and increased free water. These alterations likely contribute to the observed impairment in white matter integrity and may reflect underlying processes such as axonal loss, demyelination, and increased tissue water content.

### 4.3 Biological interpretations of connectivity and network topology changes

To determine whether structural atrophy (Figure 1 and Figure S1) led to the disruption in structural connectivity, we performed a network‐based statistical analysis. Consistent with the findings of the voxel‐based morphometry analysis (Figure S1), the network‐based statistical analysis revealed connectivity deficits in the entorhinal cortex, periaqueductal gray, and insular cortex (Figure 4A, C). These disrupted regions are implicated in distinct neural functions. The entorhinal cortex, crucial for spatial navigation and memory, is among the earliest regions affected in AD. This early involvement significantly impacts cognition. The periaqueductal gray and insular cortex are involved in pain perception and auditory processing, respectively. Structural atrophy in the periaqueductal gray^57^ and a high Aβ burden may contribute to impaired pain processing in AD patients.^58^

The left entorhinal cortex of 5xFAD;*APOE4* mice was at the center of the disrupted network connected with four edges (Figure 4A, B). These disrupted interhemispheric connections are potentially connected through the corpus callosum. This disruption in connectivity may be attributed to the reduced fractional anisotropy (Figure 2E) and neurite density (Figure 3C) in the corpus callosum of 5xFAD;*APOE4* mice.

The disrupted structural connectivity suggested that nodes failed to physically connect in 5xFAD;*APOE4* mice. To investigate information flow among nodes, we examined network properties using graph theory. Graph theory is a valuable tool for analyzing abnormalities in the organization and topology of anatomical brain networks.^59^ We identified a reduced betweenness centrality in 5xFAD;*APOE4* mice (Figure 4E). Betweenness centrality assesses a node's ability to serve as a connection between distinct clusters. Lower betweenness centrality indicates fewer “traffic hubs”, suggesting inefficient information routing with fewer direct pathways. These alterations in connectivity and network topology in 5xFAD;*APOE4* mice reveal regional network adaptations occurring in the presence of amyloid pathology, potentially driving synaptic dysfunctions in AD.

### 4.4 Cellular mechanism of white matter integrity loss

Previous human studies have shown reduced fractional anisotropy in *APOE4* carriers. However, the underlying cellular basis for this decrease in fractional anisotropy has been unclear. To address this critical question, we investigated potential mechanisms behind the reduced fractional anisotropy (Figure 2) and neurite density indices (Figure 3). We analyzed the levels of myelination and actively myelinating oligodendrocytes in *APOE3* and *APOE4* knock‐in mouse models with amyloid. We found that *APOE4* reduces myelination (Figure 5D, G), possibly leading to the observed decrease in fractional anisotropy (Figure 2H, I). Previous studies using humanized *APOE3* and *APOE4* mouse models without amyloid pathology revealed inconsistent findings regarding myelination. One study found no significant difference in myelination, while others observed reduced myelination from electron microscopy images.^27^, ^28^ These discrepancies suggest that the myelination deficits we observed might have emerged only in the presence of amyloid.

Mature oligodendrocytes are essential for the formation and maintenance of myelin sheaths around axons in the adult brain. To determine the impact of *APOE4* allele on mature oligodendrocytes, we quantified the number of them in *APOE3* and *APOE4* mouse models with amyloid pathology. 5xFAD;*APOE4* mice had a reduced number of mature oligodendrocytes in the corpus callosum compared to 5xFAD;*APOE3* mice (Figure 6F). This result aligns with a previous in vivo study that reported^27^ a reduction in CC1‐positive oligodendrocytes (a marker for mature oligodendrocytes) in the *APOE4*, even in the absence of Alzheimer's pathology. Taken together, this result suggests that a reduction in mature oligodendrocytes may contribute to myelin deficits and white matter abnormalities in the presence of *APOE4 allele*.

In addition to mature oligodendrocytes identified by CC1 antibody, actively myelinating oligodendrocytes are crucial for understanding the pathological basis of demyelination. BCAS1‐positive oligodendrocytes serve as a marker for these actively myelinating oligodendrocytes and are distinct from both oligodendrocyte progenitor cells and mature oligodendrocytes.^25^ We observed a reduction in the number of these actively myelinating oligodendrocytes in 5xFAD;*APOE4* mice. Overall, our findings suggest that the reduction in the number of both mature and actively myelinating oligodendrocytes contributes to impaired myelination and the loss of white matter integrity in 5xFAD;*APOE4* mice.

The positive correlation between MBP and fractional anisotropy (Figure S4A) demonstrates that myelin integrity is associated with coherent white matter organization. This finding is consistent with evidence that shows myelin loss is correlated with reduced fractional anisotropy in AD.^51^ Conversely, negative correlations of MBP with radial diffusivity (Figure S4B) and isotropic volume fraction (Figure S4C), as well as the positive correlation between IBA1 and radial diffusivity (Figure S4F), highlight that demyelination and neuroinflammation together may drive microstructural disorganization.^52^ In 5xFAD;*APOE4* mice, amyloid accumulation presumably exacerbates disruption of the tissue environment, damages neurites, contributing to elevated water content (Figure S4D), further pointing to white matter impairment through pathological processes.

This study provides valuable insights into the deterioration of white matter integrity in the context of amyloid pathology, but, as with all research, it also has a few limitations that need to be acknowledged. First, its cross‐sectional design precluded the identification of the exact onset time for white matter integrity changes. A future longitudinal study with multiple time points (i.e., young and middle age) could address this limitation and provide a more comprehensive understanding of disease progression. Second, we did not measure myelin content using biochemical techniques, such as western blotting, due to the difficulty of isolating pure white matter from brain tissues. However, we employed two complementary staining methods to assess myelination in the white matter. Third, we did not examine the independent effects of *APOE3/4* apart from its interactions with 5xFAD‐driven amyloid pathology. Previous human and animal studies have already established the baseline consequences of *APOE3* and *APOE4* in the absence of amyloid pathology.^60^, ^61^, ^62^, ^63^ Our focus was on the disease‐relevant interactions that remain less well understood and hold greater clinical relevance.

Despite these limitations, this study is the first to apply the advanced NODDI‐MRI approach to examine microstructural tissue integrity in an amyloid mouse model with human *APOE* isoforms. By leveraging this technique, we identified significant *APOE4*‐driven reductions in fractional anisotropy, neurite density, and structural connectivity—key metrics that recapitulate clinical white matter deficits and provide a translatable platform for therapeutic testing. Importantly, we demonstrated that the *APOE4* isoform significantly reduces white matter integrity by decreasing the number of actively myelinating oligodendrocytes. Our findings provide new evidence that these actively myelinating oligodendrocytes are key contributors to white matter impairment in AD. Given the critical role of white matter in AD pathogenesis, early prevention of white matter integrity loss may slow disease progression. Therefore, future studies need to explore the pharmacologic regulation of actively myelinating and mature oligodendrocytes in patients with *APOE4* genotype, as these cells are essential for maintaining myelin and white matter health. Together, these insights highlight the potential of oligodendrocyte‐targeted therapies and NODDI‐based biomarkers to evaluate their efficacy in vivo.

## CONFLICT OF INTEREST STATEMENT

The authors declare that the research was conducted in the absence of any commercial or financial relationships that could be understood as potential conflicts of interest. Author disclosures are available in the supporting information.

## CONSENT STATEMENT

No human subjects participated in this study; consent was not necessary.

## Supporting information



Supporting Information

Supporting Information
