# Supplementary material for: Apolipoprotein ε4 exacerbates white matter impairment in a mouse model of Aβ amyloidosis by decreasing actively myelinating oligodendrocytes
Source: Alzheimers Dement. 2025 Oct 11;21(10):e70791. doi: 10.1002/alz.70791 (PMC12514940; doi:10.1002/alz.70791)
Supplement: Supplementary file 1 — Supporting Information [file ALZ-21-e70791-s002.pdf]

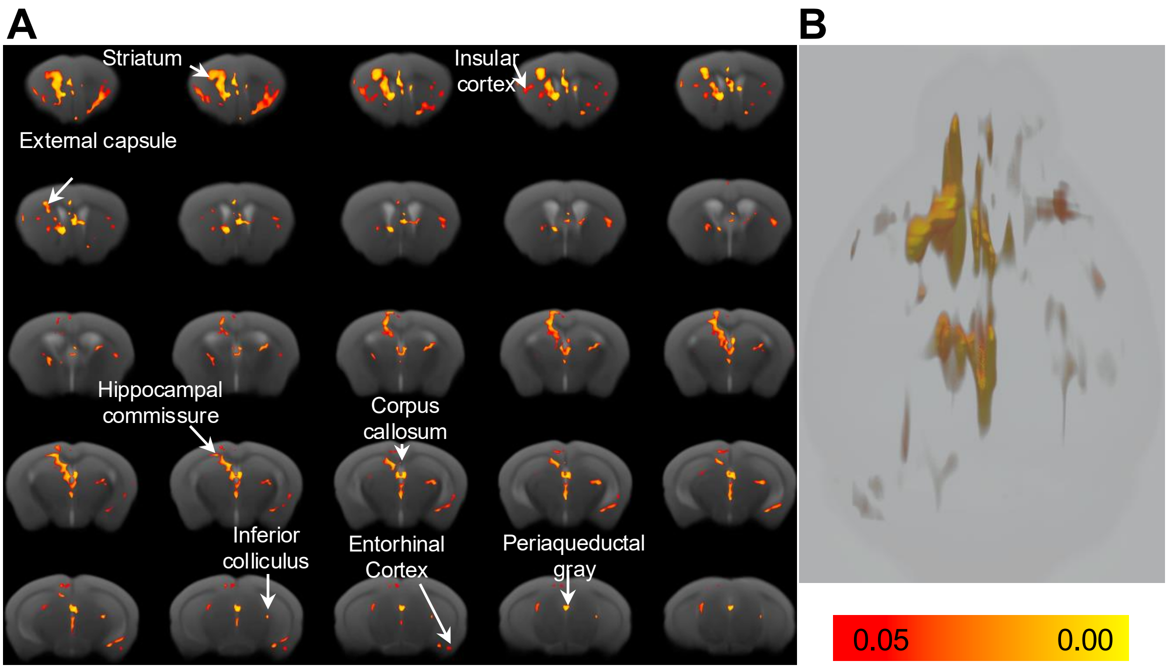

SUPPLEMENTARY FIGURE S1 Structural atrophy in 5xFAD;*APOE4* mice.
(A) Coronal slices from T_2-weighted structural brain image showing significant reduction in brain volume in 5xFAD;*APOE4* mice, compared to 5xFAD;*APOE3* mice. (B) 3D volume image of significantly atrophic clusters. Voxel-based morphometry analysis was performed with the contrast 5xFAD;*APOE3* > 5xFAD;*APOE4*. The color gradient indicates the range of statistical significance, with yellow representing the most significant regions and red representing the least significant regions. Clusters are thresholded at ($p < 0.05$) voxel level. Statistical comparisons were performed using unpaired t tests via FSL randomization for 5xFAD;*APOE3* ($n = 11$) and 5xFAD;*APOE4* ($n = 11$) mice.

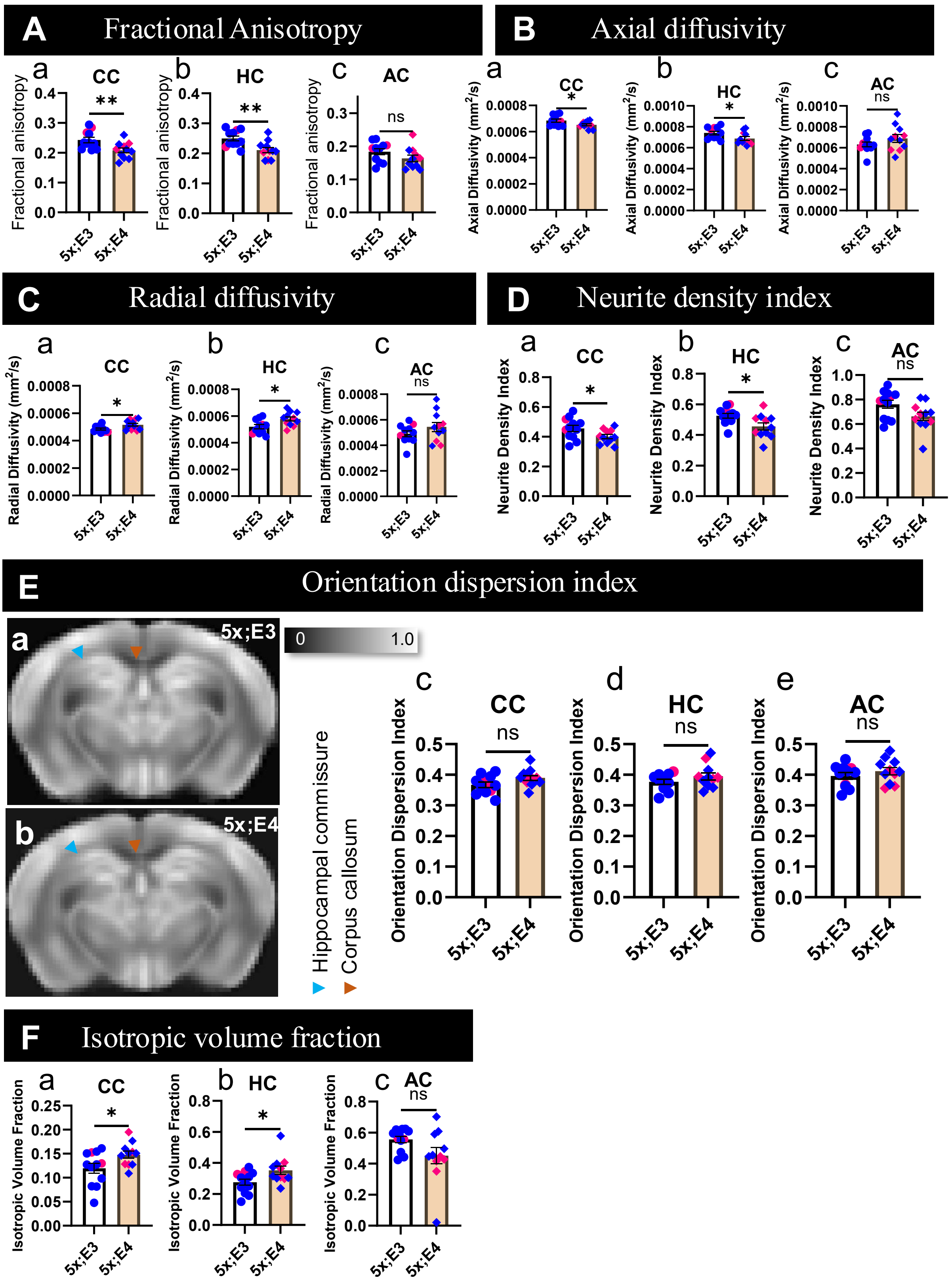

A Fractional Anisotropy
a CC **
b HC **
c AC ns
Fractional anisotropy
5x;E3
5x;E4
B Axial diffusivity
a CC *
b HC *
c AC ns
Axial Diffusivity (mm²/s)
C Radial diffusivity
a CC *
b HC *
c AC ns
Radial Diffusivity (mm²/s)
D Neurite density index
a CC *
b HC *
c AC ns
Neurite Density Index
E Orientation dispersion index
a 5x;E3
b 5x;E4
0
1.0
Hippocampal commissure
Corpus callosum
c CC ns
d HC ns
e AC ns
Orientation Dispersion Index
F Isotropic volume fraction
a CC *
b HC *
c AC ns
Isotropic Volume Fraction

SUPPLEMENTARY FIGURE S2 Atlas-based registration approach identifies impaired white matter integrity in 5xFAD;*APOE4* mice.

(A) Fractional anisotropy value for each genotype. (Aa, Ab, AC) Region specific values for corpus callosum (CC), hippocampal commissure (HC) and anterior commissure (AC). (B) Axial diffusivity value for each genotype. (Ba, Bb, Bc) Region specific values for CC, HC, and AC. (C) Radial diffusivity value for each genotype. (Ca, Cb, Cc) Region specific values for CC, HC, and AC. (D) Neurite density index value for each genotype. (Da, Db, Dc) Region specific values for CC, HC and AC. (Ea, Eb) Mean orientation dispersion index (ODI) image for each genotype. Scale bar: 0 - 1. (Ec, Ed, Ee) Tract-specific ODI values for CC, HC, and AC. (F) Isotropic volume fraction value for each genotype. (Fa, Fb, Fc) Region specific values for CC, HC, and AC. Unpaired *t*-test was performed between the groups 5x;E3 (5xFAD;*APOE3*) and 5x;E4 (5xFAD;*APOE4*). Data are presented as mean±SEM, with blue and pink bullets representing male and female mice, respectively, with n = 11/genotype. *p<0.05 and ns=not significant.

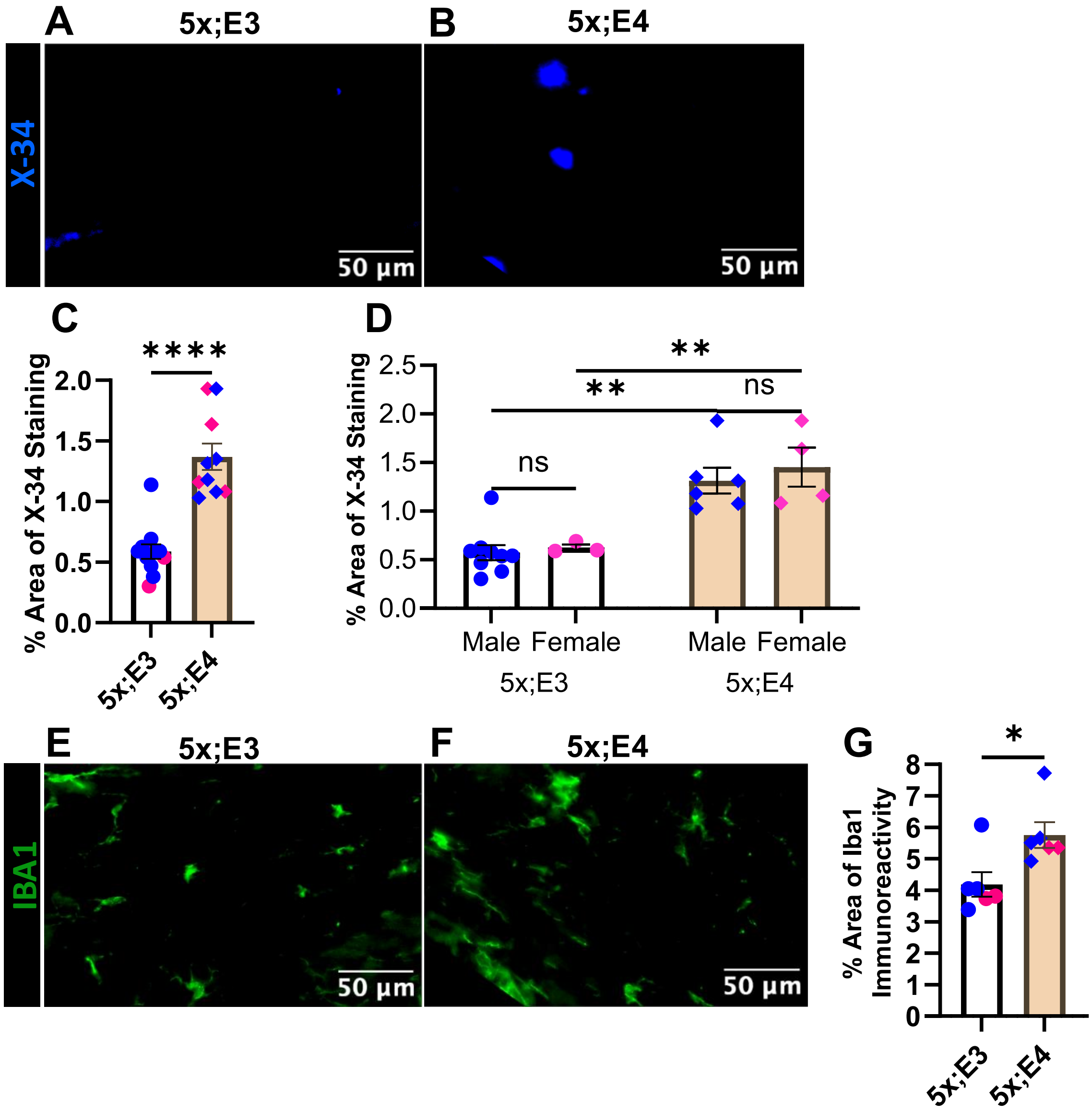

SUPPLEMENTARY FIGURE S3 Increased plaque levels and microgliosis in the corpus callosum of 5xFAD;*APOE4* mice.

(A, B) Representative X-34 stained images of fibrillar amyloid plaque in the corpus callosum for each genotype. (C) Quantification of X-34 stained (% area) for each genotype. (D) Quantification of X-34 stained (% area) by sex. (E, F) Representative IBA1-immunostained microglial images of the corpus callosum from 5xFAD;*APOE3* (E) and 5xFAD;*APOE4* (F) mice. (G) Quantification of IBA1 immunoreactive (% area) in the corpus callosum. Data are presented as the mean±SEM, Statistical comparisons were performed using unpaired *t* tests between 5xFAD;*APOE3* and 5xFAD;*APOE4* mice. Scale bar 50 µm. (C, D) $n = 12$ in 5xFAD;*APOE3* and $n = 10$ in 5xFAD;*APOE4* genotype. (G) $n = 6$/genotype. One sample from the 5xFAD;*APOE3* group was excluded from analysis due to the artificial overexposure of X-34 staining in the corpus callosum. Blue and pink markers represent male and female mice, respectively. *$p<0.05$, **$p<0.01$ and ****$p<0.0001$.

Supplementary Table 1

There were 76 nodes included in the network analysis. The nodes are listed in this table.

Sl	Node short name	Node Full name
1	Sub_L	Subiculum _L
2	Sub_R	Subiculum _R
3	Ent_L	Entorhinal cortex_L
4	Ent_R	Entorhinal cortex_R
5	Dlo_L	Dorsolateral orbital cortex_L
6	Dlo_R	Dorsolateral orbital cortex_R
7	Fra3_L	Frontal cortex, area 3_L
8	Fra3_R	Frontal cortex, area 3_R
9	Fra_L	Frontal association cortex_L
10	Fra_R	Frontal association cortex_R
11	Lo_L	Lateral orbital cortex_L
12	Lo_R	Lateral orbital cortex_R
13	M1_L	Primary motor cortex_L
14	M1_R	Primary motor cortex_R
15	M2_L	Secondary motor cortex_L
16	M2_R	Secondary motor cortex_R
17	Vmo_L	Ventromedial orbital cortex_L
18	Vmo_R	Ventromedial orbital cortex_R
19	Pa_L	Parietal association cortex_L
20	Pa_R	Parietal association cortex_R
21	S1_L	Primary somatosensory cortex_L
22	S1_R	Primary somatosensory cortex_R
23	S2_L	Secondary somatosensory cortex_L
24	S2_R	Secondary somatosensory cortex_R
25	A1_L	Primary auditory cortex_L
26	A1_R	Primary auditory cortex_R
27	A2_L	Secondary auditory cortex_L
28	A2_R	Secondary auditory cortex_R
29	Ta_L	Temporal association area_L
30	Ta_R	Temporal association area_R
31	V1_L	Primary visual cortex_L
32	V1_R	Primary visual cortex_R
33	V2l_L	Secondary visual cortex_lateral_L
34	V2l_R	Secondary visual cortex_lateral_R
35	V2ml_L	Secondary visual cortex_mediolateral_L
36	V2ml_R	Secondary visual cortex_mediolateral_R
37	Ac_L	Anterior cingulate_L
38	Ac_R	Anterior cingulate_R
39	Rs_L	Retrosplenial area_L
40	Rs_R	Retrosplenial area_R
41	In_L	Insular cortex_L
42	In_R	Insular cortex_R
43	Ect_L	Ectorhinal cortex_L
44	Ect_R	Ectorhinal cortex_R
45	Pr_L	Perirhinal cortex_L
46	Pr_R	Perirhinal cortex_R
47	Cl_L	Claustrum_L
48	Cl_R	Claustrum_R
49	End_L	Endopiriform nucleus_L
50	End_R	Endopiriform nucleus_R
51	Pir_L	Piriform nucleus_L
52	Pir_R	Piriform nucleus_R
53	Am_L	Amygdala_L
54	Am_R	Amygdala_R
55	Hp_L	Hippocampus_L
56	Hp_R	Hippocampus_R
57	Cp_L	Caudate_Putamen_L
58	Cp_R	Caudate_Putamen_R
59	Lgp_L	Lateral_Globus_Pallidus_L
60	Lgp_R	Lateral_Globus_Pallidus_R
61	An_L	Accumbens_nucleus_L
62	An_R	Accumbens_nucleus_R
63	Hyp_L	Hypothalamus_L
64	Hyp_R	Hypothalamus_R
65	Sep_L	Septum_L
66	Sep_R	Septum_R
67	Thal_L	Thalamus_L
68	Thal_R	Thalamus_R
69	Sc_L	Superior_Colliculus_L
70	Sc_R	Superior_Colliculus_R
71	Ic_L	Inferior_Colliculus_L
72	Ic_R	Inferior_Colliculus_R
73	Cb_L	Cerebellum_L
74	Cb_R	Cerebellum_R
75	Pag_L	Periaqueductal_grey_L
76	Pag_R	Periaqueductal_grey_R

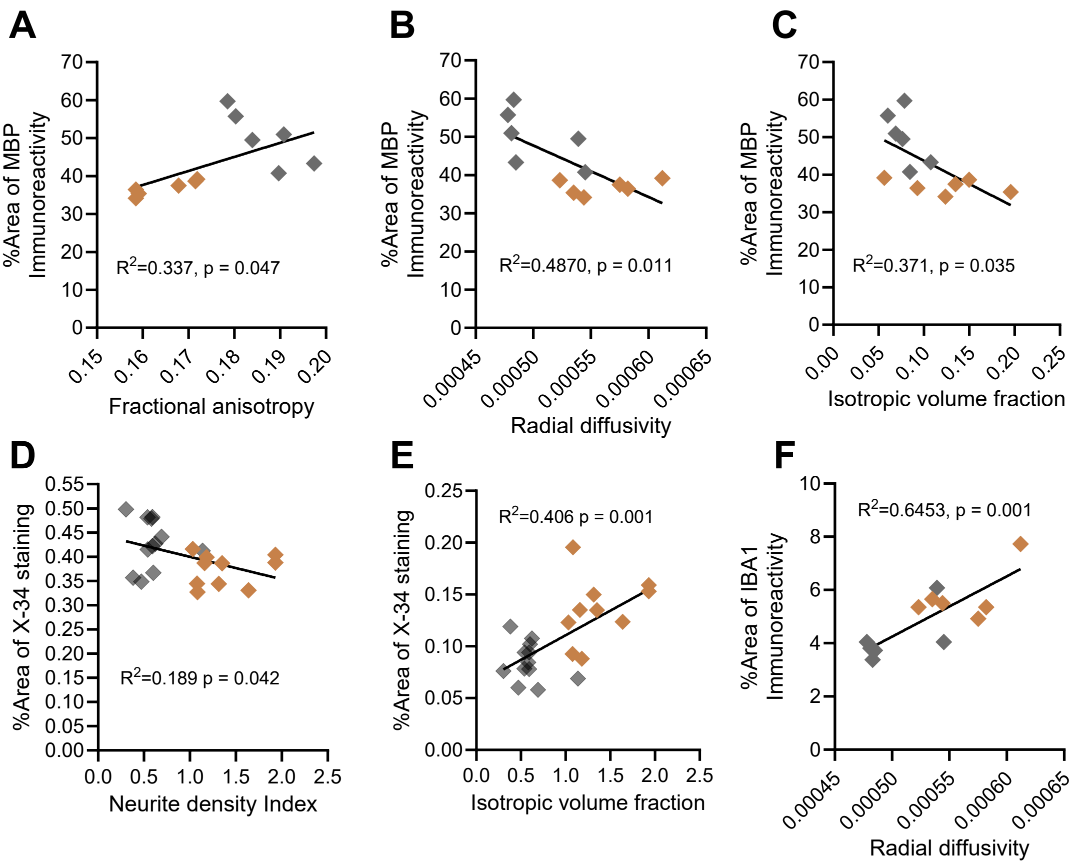

SUPPLEMENTARY FIGURE S4 Correlation between neuroimaging and histology data.

Pearson's correlations were performed between neuroimaging metrics and histology markers. (A) Correlation between the percentage area of myelin basic protein (MBP) immunoreactivity and fractional anisotropy. (B) Correlation between the percentage area of MBP immunoreactivity and radial diffusivity. (C) Correlation between the percentage area of MBP immunoreactivity and isotropic volume fraction. (D) Correlation between the percentage area of X-34-stained plaque and neurite density index. (E) Correlation between the percentage area of X-34-stained plaque and isotropic volume fraction. (F) Correlation between the percentage area of Ionized calcium-binding adaptor molecule 1 (IBA1) immunoreactivity and radial diffusivity. Gray and gold colors indicate 5xFAD;*APOE3* and 5xFAD;*APOE4,* respectively. (A-C and F) n = 6/genotype; (D-E) n = 12 in 5xFAD;*APOE3* and n =10 in 5xFAD;*APOE4* genotype. One sample from the 5xFAD;*APOE3* group was excluded from analysis due to the artificial overexposure of X-34 staining in the corpus callosum.
